# Identification and characterization of a spotted-leaf mutant *spl40* with enhanced bacterial blight resistance in rice

**DOI:** 10.1186/s12284-019-0326-6

**Published:** 2019-08-24

**Authors:** Atul Prakash Sathe, Xiaona Su, Zheng Chen, Ting Chen, Xiangjing Wei, Shaoqing Tang, Xiao-bo Zhang, Jian-li Wu

**Affiliations:** 1 State Key Laboratory of Rice Biology, China National Rice Research Institute, Hangzhou, 310006 China; 2 Nanchang Business College of Jiangxi Agricultural University, Nanchang, 330044 China

**Keywords:** Rice, Spotted-leaf, Programmed cell death, Bacterial blight, Defense response

## Abstract

**Background:**

Spotted leaf mutants show typical necrotic lesions that appear spontaneously in the absence of any pathogen attack. These mutants are often characterized to exhibit programmed cell death (PCD) and activation of plant defense responses resulting in enhanced disease resistance to multiple pathogens. Here, we reported a novel spotted-leaf mutant, *spl40* that showed enhanced disease resistance response.

**Results:**

Initially lesions appeared at leaf tips during seedling stage and gradually covered the whole leaf at the tillering stage. The lesion development was light-dependent. *spl40* showed obvious cell death at and around the lesion, and burst of reactive oxygen species (ROS) was accompanied by disturbed ROS scavenging system. Photosynthetic capacity was compromised as evidenced by significant reductions in chlorophyll content, important photosynthesis parameters and downregulated expression of photosynthesis-related genes which ultimately led to poor performance of major agronomic traits. *spl40* exhibited enhanced resistance to 14 out of 16 races of bacterial blight pathogen of rice, caused by *Xanthomonas oryzae* pv. *oryzae*, most probably though activation of SA and JA signaling pathways, owing to upregulated expression of SA and JA signaling genes, though the exact mechanism remain to be elucidated. The spotted-leaf phenotype was controlled by a novel single recessive nuclear gene. Genetic mapping combined with high throughput sequencing analysis identified Os05G0312000 as the most probable candidate gene. Sequencing of ORF revealed a single SNP change from C to T that resulted in non-synonymous change in amino acid residue from leucine to phenylalanine. Interestingly, the complementation plants did not display lesions before heading but showed lesions at the heading stage and the transgenic T_1_ progenies could be classified into 3 categories based on their lesion intensity, indicating the complex genetic nature of the *spl40* mutation.

**Conclusion:**

The results obtained here clearly show that genes related to defense and PCD were upregulated in accordance with enhanced disease resistance and occurrence of PCD, whereas the photosynthetic capacity and overall ROS homeostasis was compromised in *spl40*. Our data suggest that a novel spotted-leaf mutant, *spl40*, would help to elucidate the mechanism behind lesion development involving programmed cell death and associated defense responses.

**Electronic supplementary material:**

The online version of this article (10.1186/s12284-019-0326-6) contains supplementary material, which is available to authorized users.

## Background

Plants, during their lifecycle, are exposed to adverse environmental conditions imposed by different biotic and abiotic stresses. To combat against such adversaries, they are equipped with well-developed defense arsenal as a cope-up mechanism. Specifically during the plant innate immune response against pathogen attack, hypersensitive response (HR), a form of programmed cell death, plays an important role (Liu et al. 2005).

Lesion mimic mutants (LMMs) are so called because of the phenotype resembling the pathogen inducible, HR-mediated necrosis, which forms an important resistance mechanism in plants involving recognition of pathogens through a resistance gene-mediated defense system that induces cell death in attacked areas, which prevents further spread of pathogen to the cells in close vicinity of attack (Dangl and Jones 2001; Lam et al. 2001; Qiao et al. 2010)**.** Genetic and molecular data accumulated till date have suggested that cell death in plants associated with HR or developmental processes is genetically programmed (Lorrain et al. 2003), and this cell death is believed to be programmed cell death that plays an important role in plant growth and development (Huang et al. 2010). Several mutants exhibiting spontaneous cell death have been isolated in different plant species like maize (Wang et al. 2013), sorghum (Sindhu et al. 2018), Arabidopsis (Lorrain et al. 2003; Simanshu et al. 2014), barley (Rostoks et al. 2006), rice (Takahashi et al. 1999; Yin et al. 2000; Mori et al. 2007; Wu et al. 2008; Huang et al. 2011; Manosalva et al. 2011; Ma et al. 2012; Zhou et al. 2018)) and wheat (Kamlofski et al. 2007; Tang et al. 2013; Wang et al. 2016). As every new lesion mimic mutant is providing some useful information about plant’s cope-up mechanism at molecular as well as physiological level, the hunt for novel lesion mimic mutants is expected to continue, even at greater pace and efforts. Appearance of lesions in most of the LMM is accompanied by some defense responses like production of reactive oxygen intermediates (ROIs), accumulation of phytoalexin, callose and phenolic compounds, enhanced expression of defense marker genes and elevated levels of signaling compounds like salicylic acid (Mori et al. 2007; Qiao et al. 2010; Chen et al. 2012; Xu et al. 2018). Many LMM with disease resistance phenotype showed the elevated expression of pathogenesis-related proteins like *PR1, PR2, PR5, PR10, PBZ1* and PAL, which are the important components of plant defense against pathogens (Wang et al. 2005; Mori et al. 2007; Takahashi et al. 2007; Qiao et al. 2010; Chen et al. 2012).

To date, many LMM or spotted-leaf genes have been cloned and characterized in rice, and most of the mutants show HR-like symptoms, but with various levels of resistance to pathogens and different reaction patterns to environmental factors, indicating the presence of multiple pathways leading to HR in rice. The identified protein products encoded by spotted-leaf genes in rice include ubiquitin ligase (Zeng et al. 2004), heat stress transcription factors (Yamanouchi et al. 2002), zinc finger protein (Wang et al. 2005), clathrin associated adaptor protein complex (Qiao et al. 2010), acyltransferase (Mori et al. 2007) and splicing factor 3 subunit 3 (Chen et al. 2012). These findings indicate that numerous proteins, with distinct functions in multiple signaling pathways, are involved in the regulation of HR cell death and disease resistance (Chen et al. 2012). As the disease resistance relates to expression of defense related genes, HR and immunity, it is expected that studies on LMMs will help to provide more insights into the mechanism underlying these processes (Takahashi et al. 1999; Wu et al. 2008). In a report involving characterization of *SPL28*, it is showed that the *SPL28* gene encodes a clathrin associated adaptor protein complex1, medium subunit μ1 (AP1M1) which appeared to be involved in the regulation of vesicular trafficking, and it was suggested that *SPL28* dysfunction caused the formation of hypersensitive response (HR)-like lesions, leading to the initiation of leaf senescence (Qiao et al. 2010). One of the best studied rice LMM *spl11*, found to encode U-box/Armadillo repeat protein having E3 ubiquitin ligase activity, which showed broad-spectrum disease resistance (Zeng et al. 2004). E3 ubiquitin ligase is a well characterised component of ubiquitin-26 s proteasome system (UPS) which play an important role in the regulation of plant immune responses (Marino et al. 2012). The resistance showed by *spl11* to rice blast and bacterial blight is a non-specific resistance which is likely to be regulated by the downstream components of defense pathways, which in turn suggest that there might involve a possible cross-talks between the cell death pathway and defense pathways of blast and bacterial blight resistance (Huang et al. 2010).

Here, we identified a spotted-leaf mutant *spl40* from an EMS-induced Zhongjian100 mutant bank. This mutant exhibits necrotic lesions at four leaf stage and showed enhanced resistance to bacterial blight disease. Histochemical staining revealed the ROS accumulation and plant cell death at and around the lesion. The mutant is light sensitive and showed reduced chlorophyll content as well as disturbed photosynthetic capacity and ROS scavenging system. We also compared major agronomic traits as well as different physiological parameters that showed poor performance. The mutation is controlled by a single nuclear recessive gene located on the long arm of chromosome 5. We concluded that *spl40* is a novel spotted-leaf mutant with enhanced resistance to multiple races of *Xoo* probably through activation of JA and SA signaling pathways. This study would facilitate the elucidation of mechanism behind initiation of lesions and HR-induced PCD.

## Results

### Phenotypic performance of *spl40*

**Fig. 1:**
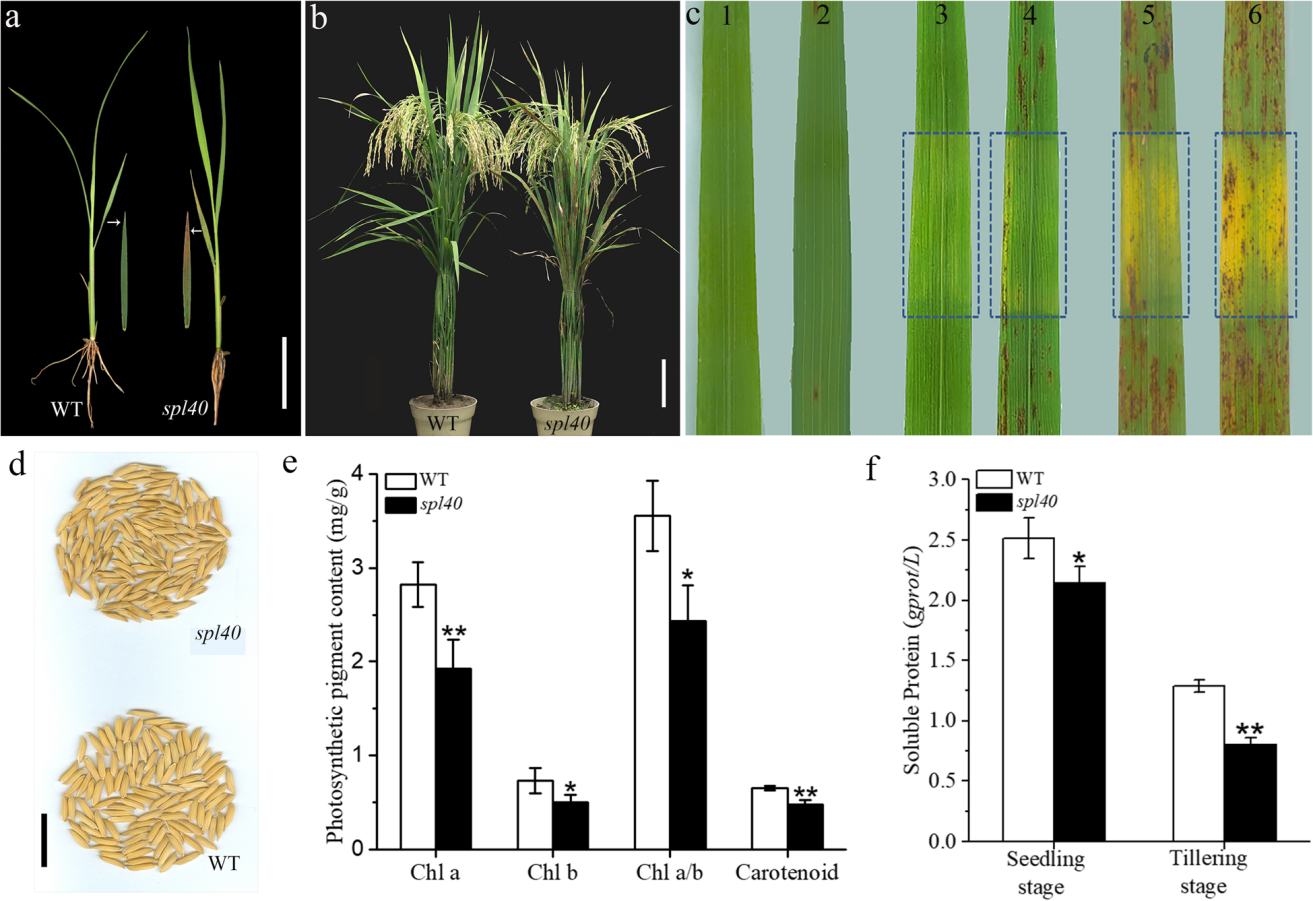
Spotted leaf phenotype of *spl40*. **a** Lesions appear at 4-leaf stage (shown detached 3rd leaf with lesions) (bar = 5 cm). **b** WT and *spl40* at maturity stage (Scale bar = 10 cm). **c** Effect of light on lesion formation under the natural condition (1–2) Zhongjian100 and *spl40* before shading. (2–3) Zhongjian100 and *spl40* shaded for 7 days. (5) *spl40* re-instated for 5 d. (6) *spl40* re-instated for 15 d. Shaded areas are boxed. **d** Lesion phenotype on matured seeds (bar = 2 cm). **e** Photosynthetic pigment contents at tillering stage. **f** Soluble protein content at seedling and tillering stage. Values are means ± SD (*n* = 3); ** indicates significance at *P* ≤ 0.01 and * indicates significance at *P* ≤ 0.05 by Student’s *t* test

Under the natural summer field conditions, leaves of *spl40* largely remained the same as the wild type (WT) till the emergence of the fourth leaf. At four leaf stage, the necrotic spots were initiated from the tip of older leaves (Fig. 1a). Later from tillering to heading, these necrotic spots became more serious and gradually spread through the whole leaf (Fig. 1b). The lesions also appeared on some husks in mutant seeds (Fig. 1d). The major agronomic traits such as number of filled grains per panicle, number of panicle per plant, seed setting rate and 1000-grain weight, were all significantly decreased in *spl40* (Table 1).

**Table 1:** Agronomic performance of *spl40*

Material	Plant height (cm)	No. tiller/Plant	No. Panicle/plant	No. filled grain/panicle	Seed-setting rate (%)	1000-grain weight (g)
WT	103.3 ± 4.2	23.3 ± 1.2	23 ± 2.0	85.7 ± 2.5	81.4 ± 6.2	21 ± 0.3
*spl40*	105 ± 2.3	21.7 ± 1.2	20.7 ± 3.6^*^	80.3 ± 2.1^*^	57.3 ± 8.9^**^	18.3 ± 0.3^**^

Values are means ± SD (*n* = 3)

^**^ indicates significance at *P* ≤ 0.01 and ^*^ indicates significance at *P* ≤ 0.05 by Student’s *t* test

Photosynthetic pigment content was measured to check the effect of lesion on it. The levels of chlorophyll a, chlorophyll b, carotenoid and Chl a/b ratio all were significantly reduced in *spl40* compared with WT (Fig. 1e). Furthermore, there was a significant decrease in the soluble protein content in *spl40* at the seedling and tillering stages compared to WT (Fig. 1f). Altogether, this data suggested that the mutation-induced formation of necrotic lesions had negative effect on photosynthetic pigment content as well as soluble protein content, which might be the cause of poor agronomic performance of the mutant.

### Lesion initiation is light-dependent

A number of lesion mimic mutants show light-dependent initiation of lesions (Huang et al. 2010; Chen et al. 2018). To determine the effect of light on lesion initiation of *spl40*, a second leaf from both WT and *spl40* was covered with 2–3 cm aluminium foil for 7 days at the tillering stage. The mutant leaf was shaded well before lesions spread to the whole leaf. The results showed that in case of *spl40*, the shaded area had nothing but very few lesions at the edge while unshaded area showed presence of numerous lesions (Fig. 1c). Later, upon re-instating the light for 5 days resulted in the initiation of lesions which were subsequently increased in number after 15 days (Fig. 1c). WT showed no obvious changes before and after the shading treatment (Fig. 1c). These results confirmed that the initiation of lesions in *spl40* was light-dependent.

### Altered photosynthetic capacity in *spl40*

**Fig. 2:**
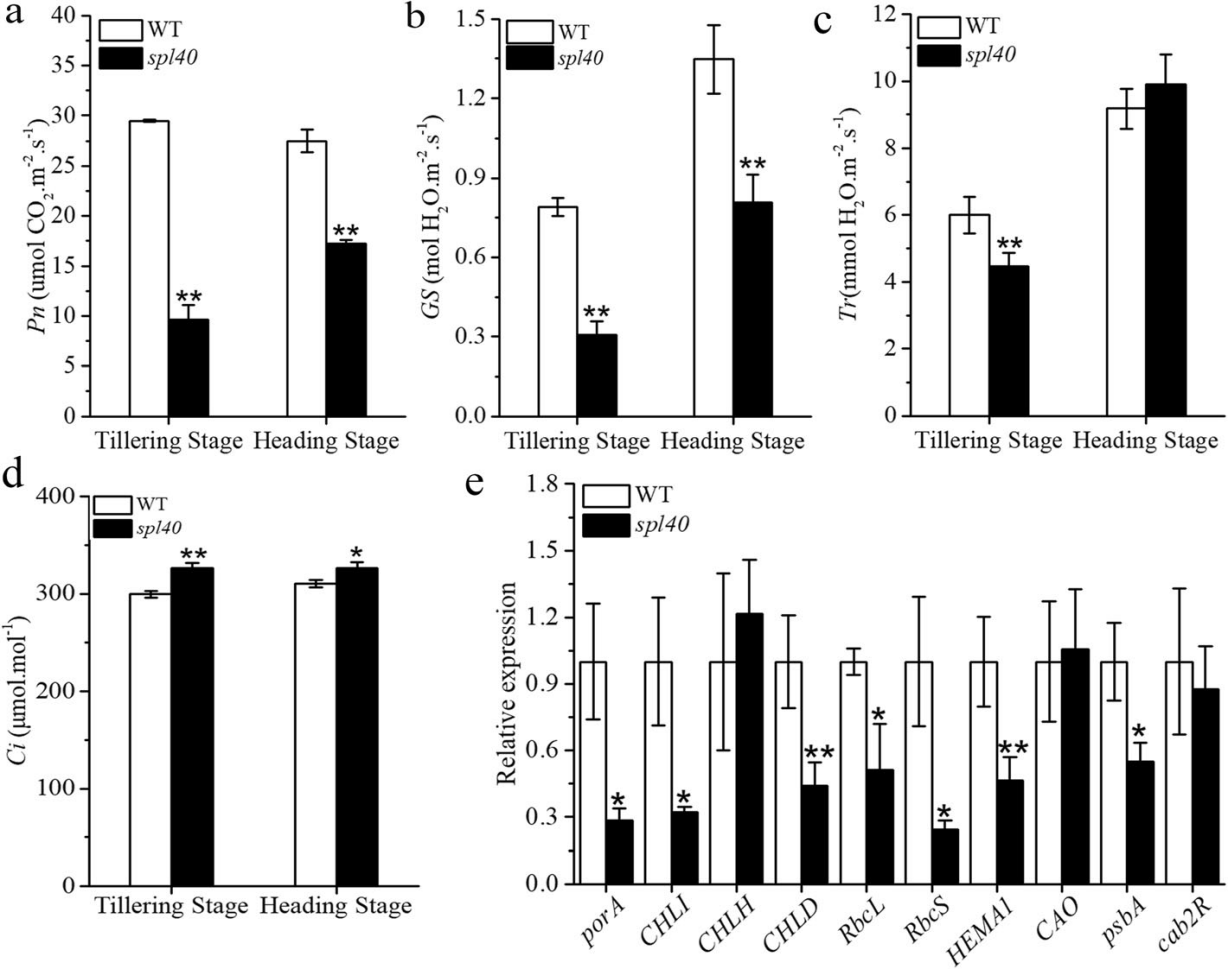
Photosynthesis parameters of leaves at the tillering and heading stages. **a** Net photosynthetic rate (*Pn*). **b** Stomatal conductance (*Gs*). **c** Transpiration rate (*Tr*). **d** Intercellular CO_2_ concentration (*Ci*). **e** Expression profile of photosynthesis related genes in *spl40* and WT at tillering stage. Values are means ± SD (*n* = 3); ** indicates significance at *P* ≤ 0.01 and * indicates significance at *P* ≤ 0.05 by Student’s *t* test

As shown above there was a reduction in the photosynthetic pigment content in the *spl40*, presence of the lesions might also lead to changes in the photosynthetic capacity. We measured the photosynthesis parameters at the tillering and heading stage in *spl40* and WT. It was found that there was a significant reduction the net photosynthetic rate (*Pn*) and stomatal conductance (*GS*) in *spl40* compared to WT at both stages (Fig. 2a, b). In case of transpiration rate (*Tr*), *spl40* showed significant reduction compared to WT at the tillering stage, whereas it was similar at the heading stage (Fig. 2c). Intercellular CO_2_ concentration (*Ci*) was significantly increased in *spl40* compared to WT at both stages (Fig. 2d). Altogether, the photosynthetic capacity was impaired in *spl40* which might be the result of reductions in photosynthetic pigment content and this ultimately led to poor agronomic performance of *spl40*.

To clarify whether the impaired photosynthetic capacity was stemmed from changes at genetic level, we measured the expression of different photosynthesis related genes in the mutant and WT at the tillering stage by qRT-PCR. The expression level of most of the photosythesis related genes was found to be significantly reduced in *spl40* compared to WT (Fig. 2e). This shows that the mutation in *spl40* has resulted in reduced expression of photosynthesis related genes which was in accordance with reduced photosynthetic pigment content and altered photosynthetic capacity.

### ROS accumulation and cell death occur in *spl40*

**Fig. 3:**
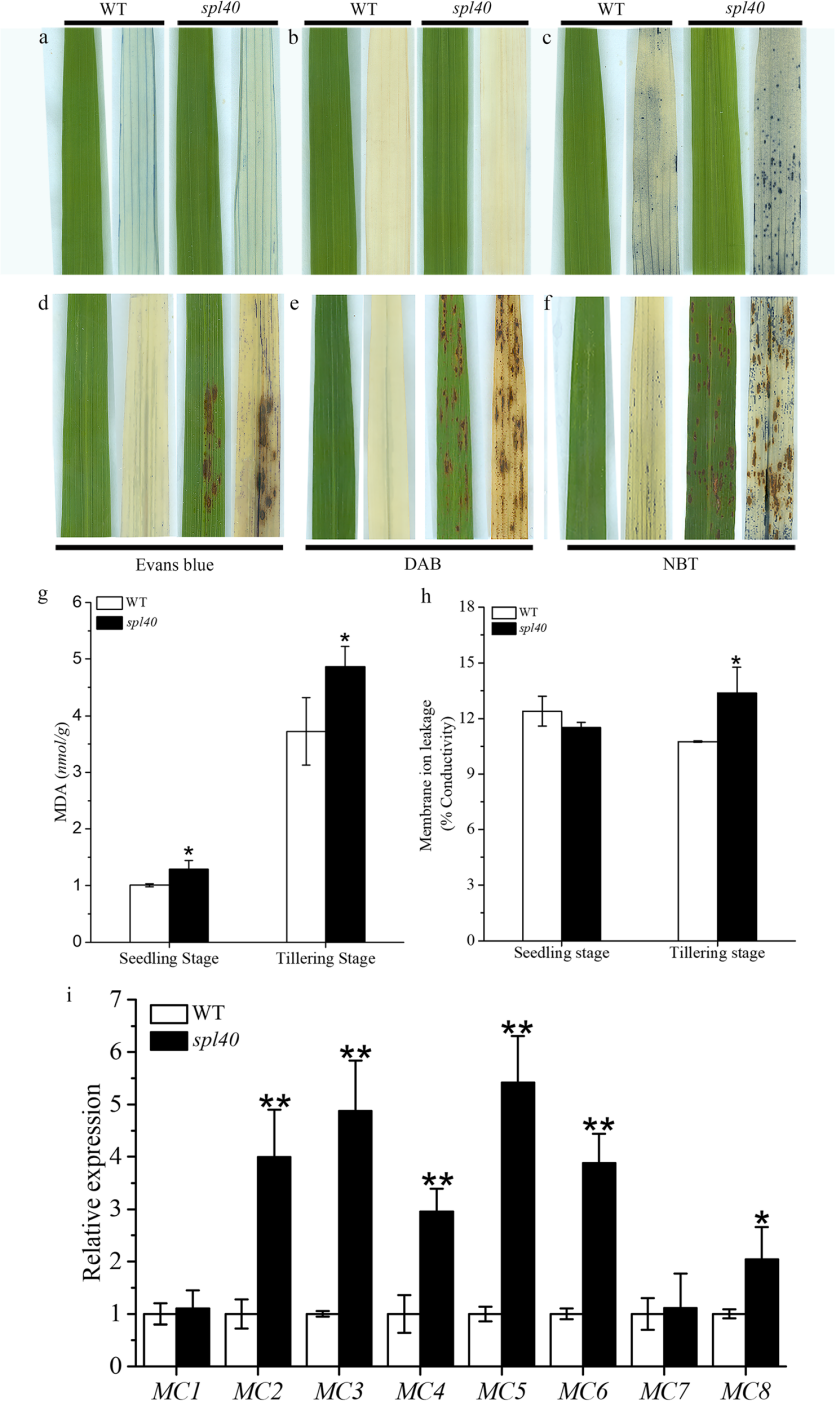
Histochemical staining of *spl40* and WT. **a** and **d** Evans blue staining: **a** before lesion development. **d** after lesion development. **b** and **e** DAB staining: **b** before lesion development. **e** after lesion development. **c** and **f** NBT staining: **c** before lesion development. **f** after lesion development. **g** Malonaldehyde (MDA) content at seedling and tillering stage. **h** Membrane ion leakage rate at seedling and tillering stage. **i** Expression analysis of PCD-related genes at tillering stage. Values are means ± SD (*n* = 3); ** indicates significance at *P* ≤ 0.01 and * indicates significance at *P* ≤ 0.05 by Student’s *t* test

To determine the occurrence of cell death as well as the accumulation of ROS at or around the lesions, we carried out histochemical staining with *spl40* and WT leaves both before the lesion development and after the lesion development. Staining with evans blue, which is an indicator of irreversible membrane damage or cell death (Liu et al. 2008), showed presence of blue stained cells in *spl40* leaves with lesion, whereas there was no staining observed before the lesion development (Fig. 3a, d). On the contrary, WT leaves were devoid of staining at both time points. To confirm the membrane damage and cell death, we further measured the levels of malonaldehyde (MDA) and membrane ion leakage. The mutant showed significantly higher levels of malonaldehyde content compared to WT both at the seedling and tillering stage (Fig. 3g), whereas membrane ion leakage was significantly higher in *spl40* than WT only at the tillering stage (Fig. 3h). Taken together these results suggested that indeed the cell death occurred in the leaves with lesions.

Further, we checked whether the occurrence of cell death in *spl40* was also accompanied by burst of reactive oxygen species (ROS). DAB staining was carried out to determine the H_2_O_2_ accumulation. The red brown precipitate was observed on *spl40* leaves with lesion (Fig. 3e), whereas no precipitate was observed on leaves without lesion (Fig. 3b). WT leaves remained clear without any precipitate both before and after the lesion development stage. NBT staining, which is the indicator of O_2_^−^ accumulation, showed blue stained regions in *spl40* leaves both before and after lesion development (Fig. 3c, f). Leaves with lesion had higher precipitate than leaves without lesion. WT leaves also showed some positive staining but it was significantly less compared to *spl40*. These results confirmed that the occurrence of cell death was accompanied by burst of ROS.

Later, we analysed the expression pattern of different rice metacaspase (OsMC) genes, which are the important regulators of programmed cell death (Huang et al. 2015). The results showed that expression levels of *OsMC2, OsMC3, OsMC4, OsMC5, OsMC6* and *OsMC8* were significantly increased compared to WT (Fig. 3i). While the expression levels of *OsMC1* and *OsMC7* were similar in *spl40* and WT. Together, our results further demonstrated that the mutation caused PCD to occur with concurrent increase in the expression of rice metacaspase genes in mutant.

### Perturbed ROS scavenging system in *spl40*

**Fig. 4:**
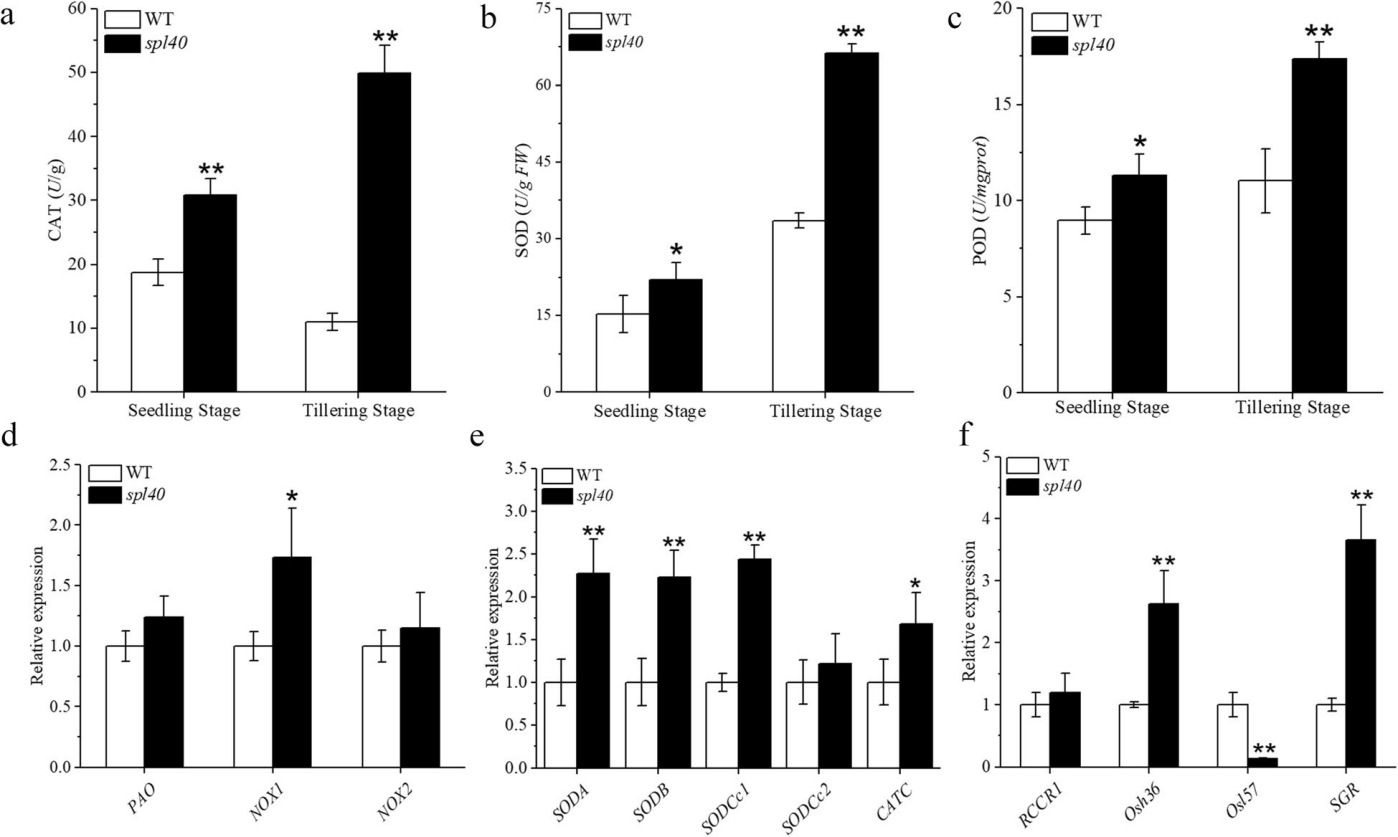
ROS scavenging enzymes and associated genes. **a**–**c** Enzymatic activities of CAT, SOD and POD at seedling and tillering stage. SOD, superoxide dismutase; CAT, catalase; POD, peroxidase. **d** Expression levels of genes encoding ROS-generating enzymes. **e** Expression levels of ROS detoxification-related genes. **f** Expression levels of senescence-related genes. Values are means ± SD (*n* = 3); ** indicates significance at *P* ≤ 0.01 and * indicates significance at *P* ≤ 0.05 by Student’s *t* test

Burst of reactive oxygen species (ROS) is commonly associated with perturbed ROS scavenging system. To check this possibility, we measured the activities ROS scavenging enzymes in *spl40* and WT leaves. The activities of CAT, SOD and POD all were significantly increased in *spl40* compared to WT both at the seedling and tillering stage (Fig. 4a–c). This clearly showed that burst of ROS resulted in perturbed ROS scavenging system in *spl40*. To further confirm this conclusion, we carried out the expression analysis of different ROS-homeostasis related genes. *NOX* (*NOX1* and *NOX2*) and *PAO* genes encode the major ROS generating enzymes NADPH oxidase (NOX) and polyamine oxidase (PAO), respectively (Lee et al. 2018). Expression of *NOX1* was significantly increased in the mutant compared to WT (Fig. 4d). As *spl40* exhibited enhanced ROS accumulation, we next examined the expression of ROS scavenging genes. Expression levels of SODA, *SODB, SODCc1* and *CATC* were significantly higher in the mutant than WT (Fig. 4e). Subsequently, we also measured the expression of important senescence related genes. *spl40* showed significantly enhanced expression of *Osh36* and *SGR* compared to WT, whereas the expression of *Osl57* was significantly decreased in the mutant (Fig. 4f). Our results demonstrated that the mutation led to increased activity of ROS scavenging enzymes along with varied changes in the expression of ROS-generating, ROS-scavenging and senescence related genes, which contributed to overall perturbed ROS homeostasis in the mutant.

### Enhanced resistance to bacterial blight pathogen

**Fig. 5:**
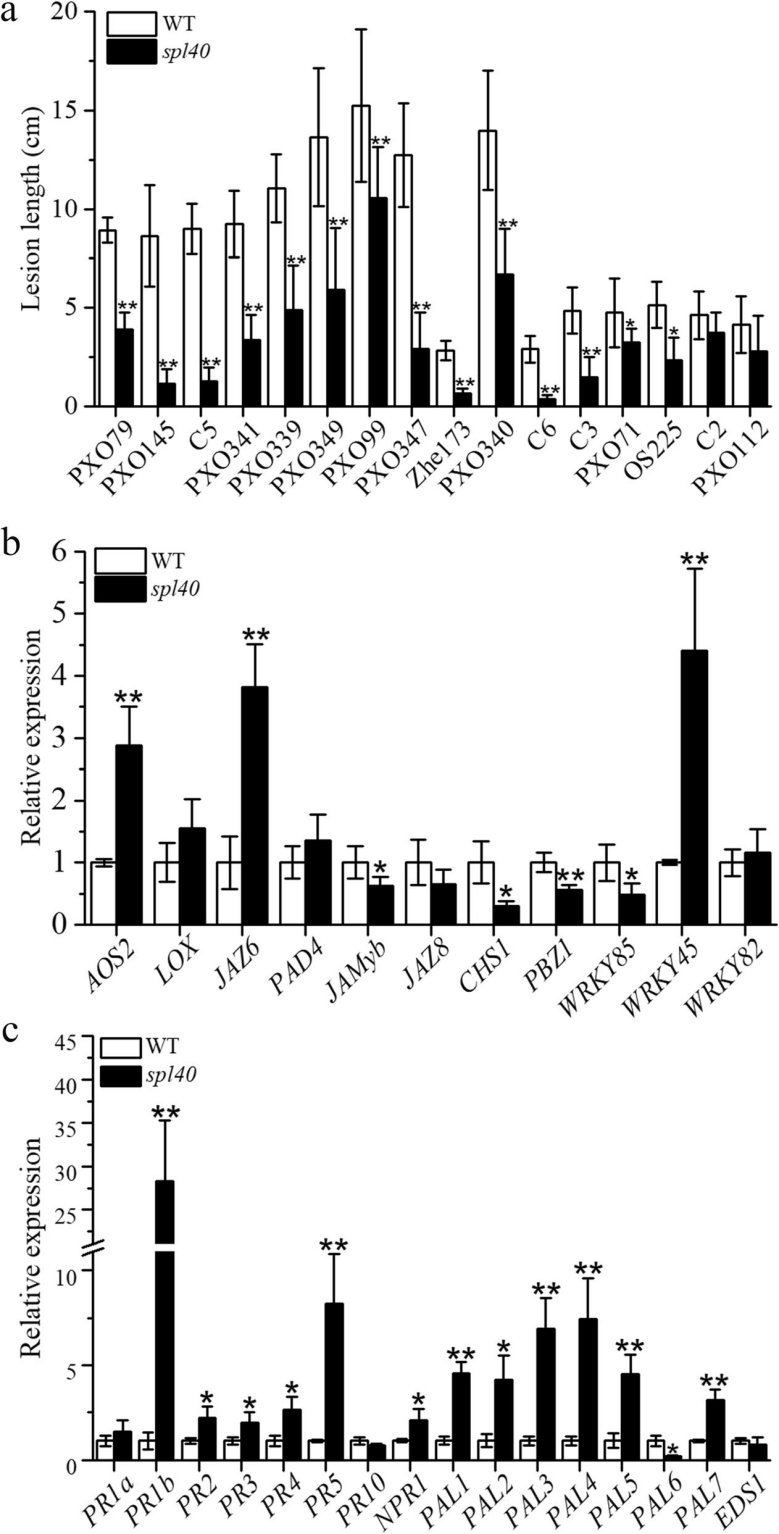
Evaluation of disease resistance to *Xanthomonas oryzae* pv. *oryzae* and expression of defense genes involved in JA and SA signaling pathway. **a** Lesion lengths after *Xoo* inoculation at 20 DPI. **b** Expression analysis of JA signaling pathway genes. **c** Expression analysis of SA signaling pathway genes. Values are means ± SD (A *n* = 9; B and C *n* = 3); ** indicates significance at *P* ≤ 0.01 and * indicates significance at *P* ≤ 0.05 by Student’s *t* test

Formation of lesions in rice LMM often lead to activation of defense responses that results in enhanced resistance to one or more pathogens (Wu et al. 2008; Feng et al. 2013; Zhang et al. 2018). We carried out the disease resistance assessment with 16 races of *Xanthomonas oryzae* pv. *oryzae* under the natural field conditions by leaf clipping method (Kauffman 1973). Top second leaf of *spl40*, WT and susceptible control IR24 were inoculated with Xoo pathogen at the tillering stage. The results showed that *spl40* exhibited significantly enhanced resistance to races PXO79, PXO145, C5, PXO339, PXO341, PXO349, PXO99, PXO347, Zhe173, PXO340, C6, C3 (*P* ≤ 0.01), PXO71 and OS225 (*P* ≤ 0.05) compared to WT, while resistance response to races C2 and PXO112 was similar in both *spl40* and WT (Fig. 5a). The IR24 showed susceptible reaction to all the races tested. The results suggested that *spl40* is a mutant with enhanced resistance to multiple races of *Xoo*.

To ascertain whether enhanced disease resistance response was stemmed from elavated expression of defence related genes, we examined their expression by real-time quantitative PCR analysis. These defense genes mainly consisted of those involved in SA and JA signalling and/or biosynthesis. Expression level of JA-signalling genes *AOS2, WRKY45* and *JAZ6* was significantly higher, while the expression of *JAMyb, CHS1*, *PBZ1* and *WRKY85* was singificantly reduced, in *spl40* compared to WT (Fig. 5b). Most of the SA-signalling genes, *PR1b, PR2, PR3, PR4, PR5, NPR1, PAL1, PAL2, PAL3, PAL4, PAL5* and *PAL7* were highly expressed in *spl40* compared to WT (Fig. 5c). This clearly showed that the elavated expression of SA signalling genes as well as some JA signalling genes might have resulted in enhanced resistance to *Xoo* in *spl40*.

### Genetic control and physical mapping of *spl40* candidate gene

To determine the genetic control of the mutation, a cross was made between *spl40* and the wild type Zhongjian100. F_1_ progenies showed normal phenotype similar to WT suggesting that mutation was recessive in nature. Further, 423 F_2_ progenies were screened for genetic segregation and found that 308 individuals exhibited normal phenotype, while 115 individuals exhibited mutant phenotype. Thus, segregation pattern fitted to 3:1 Mendelian ratio (χ^2^ = 0.62 < χ^2^_0.05_ = 3.84). Collectively, this suggested that spotted-leaf phenotype of the *spl40* was governed by a single recessive nuclear gene.

**Fig. 6:**
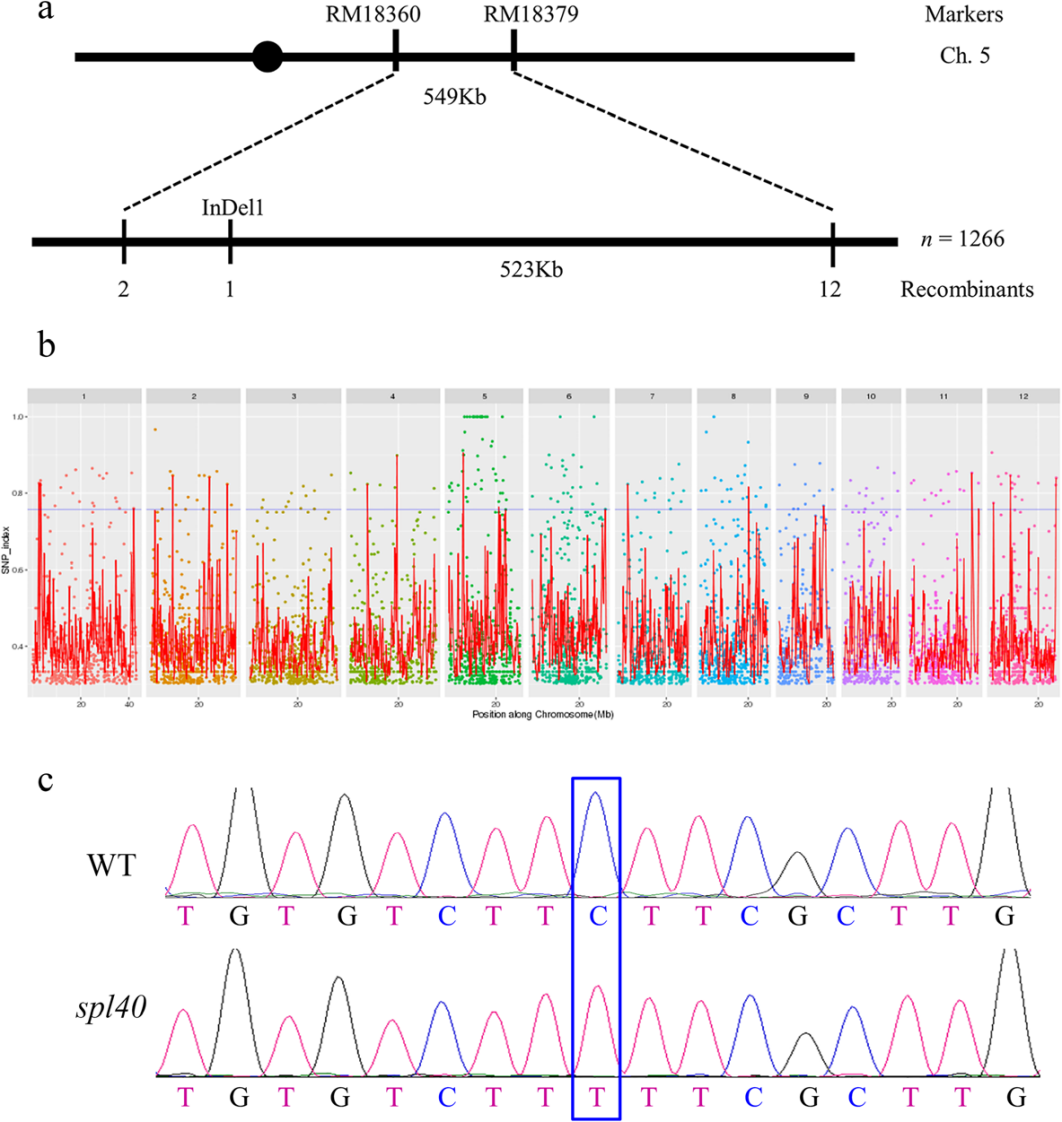
MutMap assisted mapping of *spl40* gene on the long arm of chromosome 5. **a** Fine mapping of the candidate region of *spl40*. **b** Manhattan plot depicting the distribution of SNPs along 12 rice chromosomes. **c** Confirmation of the *spl40* mutation by sequencing

An F_2_ population derived from a cross between *spl40* and Nipponbare (*Japonica*) was used to map the locus responsible for *spl40* mutant phenotype. The spotted-leaf phenotype was found to be linked with two simple sequence repeat (SSR) markers RM17952 and RM18522 on chromosome 5 by bulk segregant analysis. To fine map the locus, 1266 F_2_ individuals with *spl40* phenotype were genotyped and the mutation was finally delimited to a 523 kb interval between markers InDel1 and RM18379 (Fig. 6a).

The WT DNA and the pooled DNA from backcross F_2_ individuals were subjected to high throughput sequencing, and the results showed that more than five thousand SNPs were detected between WT and the mutant (Fig. 6b). Out of these, a total of 15 SNPs were identified with SNP index of 1 on 3 chromosomes. Among them, two were located in the 523 kb candidate region, out of which only one was present in the genic region (Fig. 6b). This resultant SNP was localized in the 10th exon Os05G0312000 which was of non-synonymous nature. A single base substitution from C to T occurred at position 2092 in the coding sequence that resulted in change of amino acid residue from leucine to phenylalanine. The mutation was confirmed by sequencing the exonic region of Os05G0312000 between the mutant and WT (Fig. 6c).

### Functional complementation with *SPL40*

**Fig. 7:**
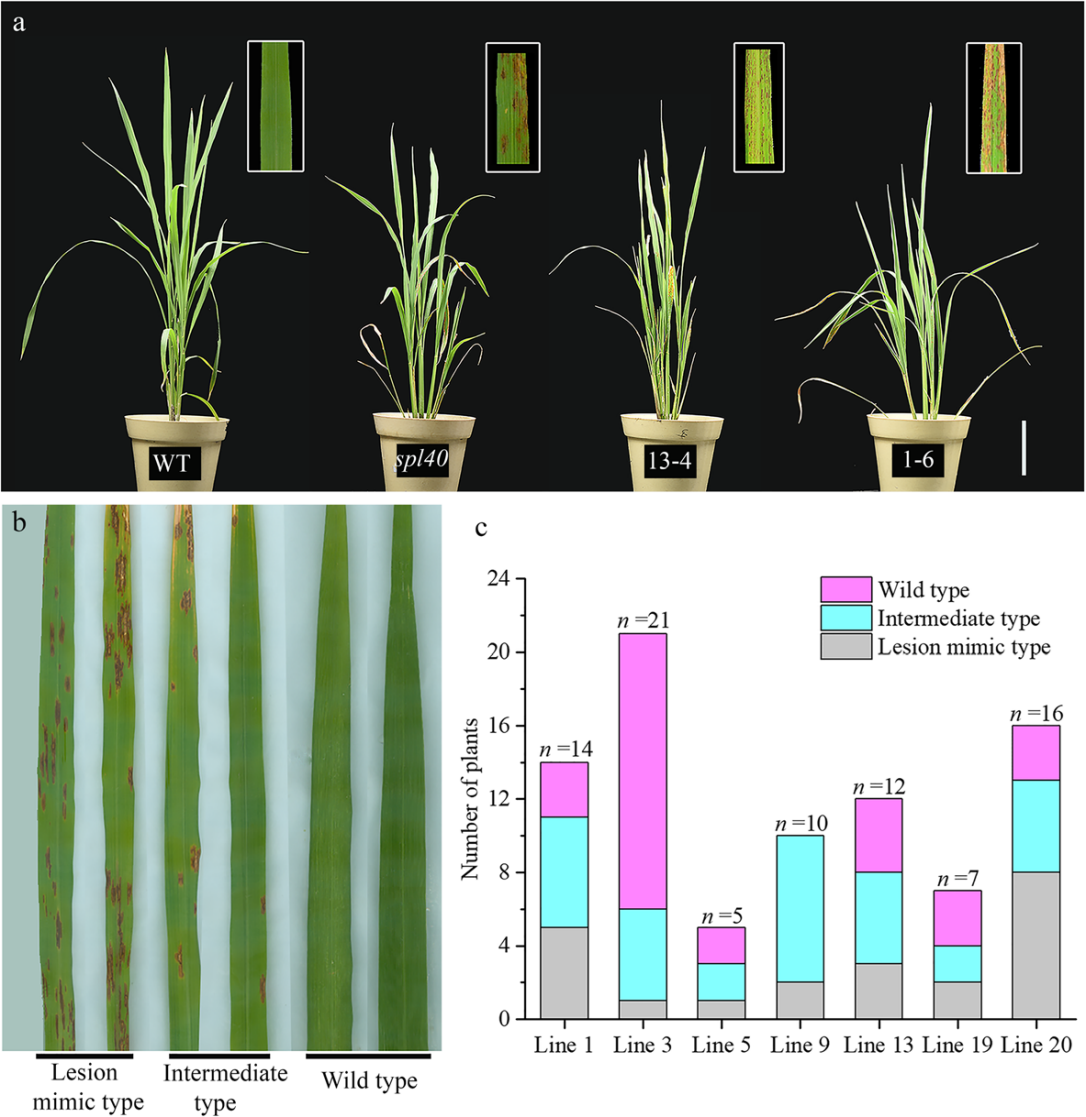
T_1_ transgenic complementation plants. **a** Phenotype of WT, *spl40* and T_1_ complementation plants (13–4 and 1–6). **b** T_1_ leaves representing three phenotypic categories viz., lesion mimic type, intermediate type and wild type. **c** Distribution of T_1_ plants in three phentoypic categories

The WT allele was transferred into the *spl40* mutant background via *Agrobacterium*-mediated plant transformation to validate the candidate gene. At the tillering stage, when *spl40* showed obvious spotted-leaf phenotype, the WT and T_0_ complementation plants did not show lesion mimic phenotype. Surprisingly, as the complementation plants entered the heading stage, brown lesions started to appear on the older leaves. At this stage *spl40* had severe spotted-leaf phenotype, while WT was normal. Further, T_1_ progenies were grown and checked for lesion mimic phenotype (Fig. 7a). It was found that, in all the lines, T_1_ plants showed different degree of phenotype severity. Based on the severity of phenotype we grouped the T_1_ plants in three categories viz., lesion mimic type, intermediate type and wild type (Fig. 7b). But in all the lines, T_1_ plants displayed complex segregation pattern (Fig. 7c). We carried out genotyping of randomly selected T_1_ plants representing each category to check for the transgene of WT allele by sequencing the region of *SPL40* that contains the mutation. We found that all the progenies showed heterozygous nature at the mutation site (Additional file 1: Table S1). Furthermore, no significance difference in the frequencies of WT allele among the three categories of T_1_ plants was detected (Additional file 2: Figure S1). The results indicate that the genetic nature of *spl40* is very complex and further work is needed to understand its genetic behavior.

## Discussion

Spotted-leaf mutants are generally considered as LMM mutants, owing to the nature of cell necrosis they show similar to that caused by hypersensitive response (HR), a type of programmed cell death (PCD) induced by pathogen invasion (Walbot et al. 1983). In present study, we have identified a novel spotted-leaf mutant *spl40* that showed lesion development similar to the HR. The mutation was controlled by a single nuclear recessive gene located on the long arm of chromosome 5. In rice, a number of spotted-leaf mutants are affected in agronomic as well as physiological processes/traits (Huang et al. 2011; Feng et al. 2013; Chen et al. 2018). For example, *Spl24*, was affected in major physiological and agronomic traits like photosynthetic pigment content, plant height, seed setting rate and 1000-grain weight (Chen et al. 2018). Similar to this, *spl40* mutant showed decrease in photosynthetic pigment content and content of soluble protein, as well as decrease in important agronomic parameters like number of filled grains per panicle, number of panicle per plant, seed setting rate and 1000-grain weight.

Till now, many LMMs have shown to exhibit light dependent appearance of lesions (Wang et al. 2015; Xiao et al. 2015). The rice *LIL1* mutant shows remarkable decrease in lesions when the light was intercepted by covering the leaf with aluminium foil (Zhou et al. 2017). Similarly, both rice *Spl24* and *HM47* spotted-leaf mutants also exhibit light-induced formation of lesions under natural light conditions (Feng et al. 2013; Chen et al. 2018). In *spl40*, lesion development was suppressed in the absence of light and appeared again when the light was re-instated. Though, at this point it is unknown which light dependent pathway is responsible for the phenomenon, but it is certain that light acts as a trigger for lesion development in *spl40*.

Lesions formed in spotted-leaf mutants are similar to the HR lesions caused upon pathogen attack. HR in response to pathogen attack involves rapid death of plant cells in vicinity of pathogen infection to prevent the further spread (Lorrain et al. 2003). Evans blue staining, an indicator of cell death, showed the occurrence of cell death after the lesion development in *spl40*. This was supported by the elevated expression of *OsMC* genes that are important regulators of PCD in plants. ROS signaling plays an important role in HR, and ROS generation is closely associated with localized cell death (Zurbriggen et al. 2010). As indicated by DAB and NBT staining, *spl40* mutant showed burst of ROS like H_2_O_2_ and superoxide (O_2_^−^). Number of reports suggests that ROS can function as a regulator of cell death (Jabs et al. 1996; Overmyer et al. 2000; Mateo 2004; Danon et al. 2005; Li et al. 2013; Kaurilind et al. 2015). *Arabidopsis* lsd1 mutants exhibited impaired control of cell death in the absence of pathogen and, superoxide was necessary and sufficient to initiate lesion formation (Jabs et al. 1996). Superoxide accumulated before the onset of cell death and subsequently in live cells adjacent to spreading *lsd1* lesions. Another *Arabidopsis* mutant, rcd1 also showed superoxide dependent propagation of lesions (Overmyer et al. 2000). In rcd1, ethylene dependent cellular superoxide accumulation occurred ahead of expanding lesions before visible symptoms appear and exogenous application of ethylene increased superoxide-dependent cell death. We speculate that higher superoxide accumulation in *spl40* before the onset of lesion might be acting as a trigger to induce cell death. *NOX1*, encoding NADPH oxidase which is one of the main ROS source, was highly expressed in *spl40*. Plants detoxify the harmful effects of hyper-accumulated ROS using ROS scavenging system comprising of important enzymes like CAT, SOD and POD. In this study, the activities of ROS scavenging enzymes were significantly higher in *spl40* mutant. Elevated expression of genes involved in ROS scavenging enzyme synthesis was in concurrent with higher activity of ROS scavenging enzymes. ROS burst along with increased activities of ROS scavenging enzymes indicate that the overall ROS homeostasis was disturbed in *spl40* mutant. In addition to this, senescence related genes were differentially expressed in the mutant as well. More importantly, the *SGR* gene was highly expressed in *spl40*, which is known to involve in chlorophyll degradation (Lee et al. 2018). This indicates that the activation of chlorophyll degradation genes might be responsible for decrease in chlorophyll level in *spl40*.

Spotted-leaf mutants often show enhanced resistance to pathogen infection along with constitutive expression of defense response genes (Yin et al. 2000; Mizobuchi et al. 2002; Wu et al. 2008; Xu et al. 2018). In a rice spl21 mutant, upregulated expression of pathogenesis-related genes and increased level of jasmonic acid leads to enhanced resistance to bacterial blight pathogen *Xanthomonas oryzae* pv. *oryzae* (Xu et al. 2018). In present study, *spl40* mutant showed enhanced resistance to 14 races of this pathogen. This was supported by upregulated expression of important genes involved in JA and SA signalling pathways. Therefore we conclude that *spl40* confers enhanced resistance to bacterial blight pathogen through activation of SA and JA metabolic pathway. But the specific mechanism and the factors involved in the resistance remain to be identified, and this will lead to the future course of this research.

MutMap is a powerful tool that employs whole genome resequencing approach to rapidly identify the causal mutations (Abe et al. 2012; Fekih et al. 2013; Takagi et al. 2013, 2015). Genetic mapping combined with MutMap analysis revealed that Os05G0312000 harbored a single base substitution in the 10th exon that resulted in change in the amino acid residue from leucine to phenylalanine. Although, we could not test the expression of large number ORFs present in the mapping region (about 101 ORFs in 523 kb region), based on high-throughput sequencing results we considered that Os05G0312000 is the most likely candidate gene and decided to carry out functional complementation test. Functional complementation with WT allele was carried out and the T_0_ transgenic plants did not develop the phenotype till the tillering stage, while *spl40* showed the phenotype at the seedling (four leaf) stage. Unexpectedly, the lesion mimic phenotype was observed at the beginning of the heading stage in the T_0_ plants. Further, T_1_ progenies consisted of three categories based on the phenotype viz., lesion mimic type, intermediate type and wild type. Though, through functional complementation, we confirmed that Os05G0312000 was likely the correct candidate gene, its genetic nature seems complex. In silico search in Rice Annotation Project Database (http://rice.plantbiology.msu.edu/) showed that Os05G0312000 is predicted to function as a subunit (Med5_1) of the Mediator complex in Arabidopsis. Mediator is a master transcriptional regulator in eukaryotes that serves as a molecular bridge between gene-specific transcription factors bound at enhancers, and RNA polymerase II (RNA pol II) (Flanagan et al. 1991; Carlsten et al. 2013; Buendía-Monreal and Gillmor 2016). It consist of 25 subunits in yeast and approximately 34 subunits in plants, which reside in four distinct modules, termed head, middle, tail and CDK8/kinase (Samanta and Thakur 2015). Bäckström et al. (2007) successfully carried out the purifcation of Mediator complex in *Arabidopsis* and showed that specific plant Mediator subunits were linked to the regulation of specialized processes such as the control of cell proliferation and the regulation of flowering time in response to light quality. In plants, Mediator has been found to play important roles in diverse aspects of plant life right from growth and development upto the response against biotic and abiotic stresses (Samanta and Thakur 2015). In the present study, we figure that dysfunction of the Mediator indirectly leads to disturbed ROS homeostasis and upregulated expression of cell death related genes which might finally contribute to the occurrence of cell death in *spl40*. However, the T_1_ heterozygotes with a similar expression level of WT allele displaying various intensity of lesions are yet to be further invertigated. Furthermore, the complicated genetic nature of *spl40* is also evident from the fact that an allelic Nipponbare mutant *spl40*^*NIP*^ with single base substitution in the same locus (Os05G0312000) exhibited different phenotype compared with *spl40* (Additional file 2: Figure S2). We are considering that the locus may possess complicated biological function possibly associated with genotypes because of the diverse phenotypes. Our laboratory is currently examining the differences in background by exploring the differences in *spl40* allele in different cultivars.

## Conclusions

Our study demonstrated that *spl40* was a novel spotted leaf mutant compromised in number of traits such as poor agronomic performance with altered photosynthetic capacity, HR-like cell death and disturbed ROS homeostasis. In addition, *spl40* showed enhanced disease resistance to bacterial blight and immunity-associated senescence. This study would facilitate the efforts to unravel the mechanism of lesion development involving programmed cell death and defense responses.

## Methods

### Plant materials and growth conditions

A spotted-leaf mutant *spl40* was isolated from an ethyl methane sulfonate (EMS)-induced mutant bank of Zhongjian100, an indica rice. The mutant, Zhongjian-100 (wild-type, WT) and F_2_ populations were grown in the paddy field in summer of 2016 at China National Rice Research Institute (CNRRI) in Hangzhou, Zhejiang, China. The back-cross F_2_ population derived from the cross *spl40*/Zhongjian-100 was used for genetic analysis and the *spl40*/Nipponbare derived F_2_ population was used for gene mapping. The WT and *spl40* were grown for evaluation of agronomic traits including plant height, tiller number, number of panicle/plant, number of filled grain/panicle, seed setting rate and 1000-grain weight. Agronomic traits were recorded at maturity stage on three individual plants, and the means from three replicates were used for analysis.

### Histochemical analysis

Leaf samples from WT and *spl40* were collected at the tillering stage. Evans blue staining and nitro blue tetrazolium (NBT) staining was performed to determine the occurrence of cell death and accumulation of superoxide (O_2_^−^) (Liu et al. 2008; Qiao et al. 2010). The detection of H_2_O_2_ was carried out by using 3, 3′-Diaminobenzidine (DAB) staining as previously described (Thordal-Christensen et al. 1997).

### Shading experiment

At the tillering stage, the top second leaf of WT and *spl40* was used to carry out the shading experiment. The mutant leaves in which lesions have started to appear at the tip, were shaded with a piece of 2–3 cm foil for 7 days under natural light conditions. The foil was then removed and light was reinstated for 5–15 days to investigate the influence of natural light on the initiation of lesions. Leaves were photographed by a scanner.

### Measurement of physico-biochemical parameters

The contents of Chlorophyll a (Chl a), chlorophyll b (Chl b) and carotenoid were determined according to He et al. (2018). The contents of malonaldehyde (MDA) and soluble protein, and the enzymatic activities of peroxidase (POD), superoxide dismutase (SOD) and catalase (CAT) were measured following the manufacturer’s instructions (Nanjing Jiancheng Bioengineering Institute, Nanjing, China). Enzymatic activity analysis of POD, SOD, CAT and measurements of soluble protein and MDA content were carried out at the seedling and tillering stages. Photosynthesis related parameters such as net photosynthesis rate (*Pn*), stomatal conductance (*Gs*), intercellular CO_2_ concentration (*Ci*) and transpiration rate (*Tr*) were measured in the field by using portable L-6400XT (LI_COR, Lincoln, NB, USA). Means of three replicates were used for analysis. The membrane ion leakage measurement was carried out according to He et al. (2018). The means from three measurement were used for analysis.

### RNA extraction and gene expression analysis

The total RNA was isolated from the leaves of *spl40*, T_1_ complementary plants and WT using NucleoZOL Reagent Kit according to the manufacturer’s instructions (MACHERY-NAGEL, Düren, Germany). RNA samples were treated with DNase (Promega), and first strand copy of DNA (cDNA) was synthesized using 1 μg of RNA. Real-time fluorescent quantitative PCR was carried out using SYBR Premix ExTag™II (Tli RNaseH plus) master mix (Takara, Kusatsu, Japan) and performed on Thermal Cycler Dice® Real Time System (Takara, Kusatsu, Japan). The rice *Ubiquitin* was used as the internal control to normalize expression levels. Three biological repeats were used to obtain the final results. The relevant sequences of primer sets used in different experiments are listed in Additional file 1: Table S2. To determine the expression level of WT allele in the three categories of T_1_ complementary plants (WT, intermediate, and lesion mimic type), the m40-D primer pairs (m40-D F: GCTGCATGCTCTCATATC, m40-D R: GGAAATGAGTCAATATACA) located at exon 9 and exon 10 respectively were used to amplify the cDNA region covering the mutation site, and the RT-PCR products were cloned into the vector pMD18-T. A total of 75 colonies (3 replicates with 25 colonies/replicate) from each category of T_1_ plants were selected and used for sequencing. The frequncy of WT allele was used to determine the expression level of WT allele in T_1_ plants. Means of three biological replicates were subjected to one-way ANOVA followed by the Duncan multiple range test.

### Disease resistance evaluation

Rice plants of *spl40*, WT and susceptible control IR24 were inoculated with 16 races (PXO79, PXO145, C5, PXO339, PXO341, PXO349, PXO99, PXO347, Zhe173, PXO340, C6, C3, PXO71, OS225, C2 and PXO112) of *Xanthomonas oryzae* pv. *oryzae* (*Xoo*) by the leaf-clipping method (Kauffman 1973) at the tillering stage. The bacterial cultures were separately suspended in distilled water and adjusted to OD600 = 1.0 for inoculation. Pathogen inoculation was carried out on fully expanded second youngest leaf. Three individual plants and three leaves per plant were inoculated for each race. Disease evaluation was carried out 20 days after inoculation by measuring the legion length. Means from nine leaves were used for analysis.

### Genetic analysis and gene mapping

The F_1_ plants from the cross between *spl40*/Zhongjian100 were grown in the paddy field at CNRRI to determine the genetic nature (dominant/recessive) of *spl40*. The subsequent F_2_ individuals derived from selfing were used for segregation analysis. To map the mutation, a population derived from the cross *spl40*/Nipponbare was used. Equal amount of leaf tissues from 10 WT and 10 mutant plants were collected for DNA extraction to make the respective DNA pools. Bulk segregant analysis using the DNA pools were used to rapidly map the mutation. The F_2_ individuals with mutant phenotype were used for fine mapping of the mutation. Plant genomic DNA was isolated according to the previous protocol (Edwards et al. 1991). Simple sequence repeat (SSR) markers were obtained from the website (http://www.gramene.org/). For the insertion/deletion (InDel) markers, the genomic seqeunces of japonica cultivar Nipponbare and indica cultivar 9311 from the public databases RGP (http://rgp.dna.affrc.go.jp/E/toppage.html), Gramene (http://gramene.org/genome_ browser/index.html) and the Gene Research Center of the Chinese Academy of Sciences (http://rice.genomics.org.cn/rice/index2.jsp) were compared and markers were designed by using NCBI Primer-BLAST (https://www.ncbi.nlm.nih.gov/tools/primer-blast/). The primers were synthesized by Sangon Biotech Co. Ltd. (Shanghai, China). PCR reaction and detection were carried out as described previously (Feng et al. 2013). The relevant primer sequences for gene mapping are listed in Additional file 1: Table S3.

For the high thoughput sequencing by a modified MutMap approach, *spl40* was back-crossed to Zhongjian100 and then self crossed to generate BC_1_F_2_ population. The WT DNA and the pooled DNA of 25 mutant type individuals were used for high throughput sequencing analysis with 25 average read depth to determine SNPs between WT and the mutant (AnoRoad Genome, Beijing). The SNP index is calculated based on the frequency of mutant type SNP and the SNP with index of 1 is considered as the candidate SNP (Abe et al. 2012).

### Vector contruction for functional complementation

The genomic DNA sequence containing the entire coding sequence *SPL40* (12,419-bp), including 1966-bp upstream and 1392-bp downstream sequence was divided into four parts so as to clone it into pCAMBIA1300 vector. The four fragments were amplified using respective primers (Additional file 1: Table S4) and cloned into pMD19T(simple) vector. After sequencing, the fragments were sequentially cloned into the pCAMBIA1300 vector. Trangenic plants were developed via *Agrobacterium*-mediated plant transformation and regeneration (Hiei and Komari 2008).

## Additional files

Additional file 1: **Table S1.** Genotype of the transgene of WT allele in T_1_ progenies from three lines. **Table S2.** RT-PCR primers. **Table S3.** Markers used for fine mapping and sequencing of *spl40* locus. **Table S4.** Primers used for making complementation construct. (DOCX 31 kb)

Additional file 2: **Figure S1.** Frequency of WT *SPL40* allele in the T_1_ generation of complementary plants. Wild type (W), intermediate type (I) and Lesion mimic type(L). Values are means ± SD of three biological repeats and re subjected to one way ANOVA followed by the Duncan multiple range test. **Figure S2.** Phenotype of *spl40* and *spl40*^*NIP*^. (a) Phenotype of *spl40*; (b) Phenotype of *spl40*^*NIP*^. (PDF 255 kb)

## Data Availability

Not applicable.

## Abbreviations

APX: Ascorbate peroxidase
CAT: Catalase
Chl a: Chlorophyll a
Chl b: Chlorophyll b
*Ci*: Intercellular CO_2_ concentration
DAB: 3,3′-Diaminobenzidine
*Gs*: Stomatal conductance
HR: Hypersensitive response
InDel: Insertion/deletion marker
JA: Jasmonic acid
LMM: Lesion mimic mutant
MDA: Malonaldehyde
NBT: Nitro blue tetrazolium
NOX: NADPH oxidase
ORF: Open reading frame
PAO: Polyamine oxidase
PCD: Programmed cell death
*Pn*: Net photosynthesis rate
POD: Peroxidase
ROS: Reactive oxygen species
SA: Salicylic acid
SNP: Single nucleotide polymorphism
SOD: Superoxide dismutase
*spl40*: Spotted-leaf mutant 40
SSR: Simple sequence repeat marker
*Tr*: Transpiration rate
WT: Wild type
*Xoo*: *Xanthomonas oryzae* pv. *oryzae*
