# Supplementary material for: Identification and characterization of a spotted-leaf mutant spl40 with enhanced bacterial blight resistance in rice
Source: Rice (N Y). 2019 Aug 24;12:68. doi: 10.1186/s12284-019-0326-6 (PMC6708518; doi:10.1186/s12284-019-0326-6)
Supplement: Supplementary file 1 — Table S1. Genotype of the transgene of WT allele in T1 progenies from three lines. Table S2. RT-PCR primers. Table S3. Markers used for fine mapping and sequencing of spl40 locus. Table S4. Primers used for making complementation construct. (DOCX 31 kb) [file 12284_2019_326_MOESM1_ESM.docx]

Additional file 1: Table S1: Genotype of the transgene of WT allele in T_1_ progenies from three lines

| Line | Progeny | Genotype | Phenotype |
| --- | --- | --- | --- |
| Line 1 | 1 | C/T | I |
|  | 2 | C/T | W |
|  | 3 | C/T | L |
|  | 4 | C/T | W |
|  | 5 | C/T | I |
|  | 6 | C/T | W |
| Line 9 | 1 | C/T | W |
|  | 2 | C/T | I |
|  | 3 | C/T | I |
|  | 4 | C/T | I |
|  | 5 | C/T | I |
| Line 13 | 1 | C/T | I |
|  | 2 | C/T | L |
|  | 3 | C/T | W |
|  | 4 | C/T | L |

I: Intermediate type; L: Lesion mimic type; W: Wild type

Additional file 1: Table S2: RT-PCR primers.

| Gene | Sense primer (5′–3′) | Antisense primer (5′–3′) |
| --- | --- | --- |
| *Ubiquitin* | CCCTCCACCTCGTCCTCAG | AGATAACAACGGAAGCATAAA AGTC |
| *porA* | ATCACCAAGGGCTACGTCTC | GAGTTGTTGTTCCAGCTCCA |
| *CHLI* | CGGAGTAACCTTGGTGCTGT | CTTGGCAGCCCTGTTAGTCA |
| *CHLH* | TGACTCAGACCCGACAAAGC | TCCCCTCGTACCACTTAGGG |
| *CHLD* | GGAAAGAGAGGGCATTAG | CAATACGATCAAGTAAGTGTT |
| *RbcL* | ATCGTGCTCGCGGTATCTTT | ACCAGGTGCATTACCCCAAG |
| *rbcS* | CCGTGAGAACCACAGATCCC | ACGTTGTCGAAGCCGATGAT |
| *HEMA1* | ACACGCCATCTGTTTGAGGT | CAAGCCTCCACTGTTTTGCC |
| *CAO* | TTGGCACAAATGGAGACCC | GCTGCACTGGACCAGACAC |
| *psbA* | TGTAGCTGGTGTATTCGGCG | ATAACCATGAGCGGCCACAA |
| *cab2R* | GTTCTCCATGTTCGGCTTCT | GACGAAGTTGGTGGCGTAG |
| *RCCR1* | CGCATTTCCTCATGGAATTT | CTTCTCACGCTGTTTGTCCA |
| *Osh36* | AACGCATTTGTGGTTGGCTC | TCAACTTTGGCCGGTGTCTT |
| *Osh57* | ACCCTAAAGTAAATGAAGTC | CCTGCTCTTGTCTTGTTA C |
| *SGR* | AGGGGTGGTACAACAAGCTG | GCTCCTTGCGGAAGATGTAG |
| *OsMC1* | GCTTCATCAAGGCGGTGGAGTG | AAGTTGGCGACCTTGCGGATG |
| *OsMC2* | CGACCCGTACAGGGTGCCGA | GCACAGCGCCTCGTCGTAGC |
| *OsMC3* | GGCTCCTTCGTCCGCAAGAT | CACAGGAGAAACGGTTTCCTGT |
| *OsMC4* | TCGACGTTCGTGGAGATGCTC | ATTCACGAGCCGCCTGATCTT |
| *OsMC5* | GTGCCAGACCGACCAGACAT | CCGCTCTTCTCCGACAGGAT |
| *OsMC6* | CCACACCGCAGGGTTCTTCAT | GTCCAGGCTGCTGAGTGTATCC |
| *OsMC7* | ATACAGACCGTGCTGGCGTC | AGGAATGGCGTCTCGGCGTT |
| *OsMC8* | TCCGGCAAGTGCCTCGTAAC | CAATGCGGTCGGTCACAGGAT |
| *PAO* | CCTAGCCAAGAAGTGTTGCC | TCGCTCCCATGAAGACCTTT |
| *NOX1* | AGGCCGACTGCTTCCTCT | CACTGACAATTGCAGCAGGT |
| *NOX2* | ACTGCTTCCTCTTCGCCTCT | CTCTGTCAGCCCAGCAGTT |
| *SODA* | ATCTGGATGGGTGTGGCTAGCTTT | AGTACGCATGCTCCCAGACATCAA |
| *SODB* | TCCGCCGTATAAACTTGATGCCCT | TGGGTTGCCGTTGTTGTATGCTTC |
| *SODCc1* | GTGCATGCCGATCCTGATG | CTGGGAGATGGAAGGTGAGT |
| *SODCc2* | TGTGACGGGAAGTGTCTCTG | AGTAAGGGGGATCTGGCTGT |
| *CATC* | CCACGAGATCAGGAGCATCT | TCCGTGACTGAAGCAGATTG |
| *AOS2* | CTCGTCGGAAGGCTGTTGCT | ACGATTGACGGCGGAGGTT |
| *LOX* | GATGGCGGTGCTCGACGTGCT | GCACCTGTTCTTGAGCTTTCTAT |
| *JAZ6* | GGACATGCCGATCGCGAGGAA | GCGCGAGTGCATGTGTCCAA |
| *PAD4* | CCAACATGTACCGCATCAAG | GGTTGTTTCGGTGGTAGTGGC |
| *JAMyb* | CCGAGCATGGTGACTAGCTCATCTT | CCTTGCACCCAACCGTTAAGCTGTT |
| *JAZ8* | CCAAACACGGCGGAAACAG | GGTGGACGGGAAGTTCTCAAAG |
| *CHS1* | GACTACCCGGACTACTACTTCA | CTTCCTGATCTGCGACTTG |
| *PBZ1* | CCCTGCCGAATACGCCTAA | CTCAAACGCCACGAGAATTTG |
| *WRKY85* | CAGCAAGAAAAGGAATATACAAAT | CTCAATGTGTTTCCTAACATTACA |
| *WRKY45* | TTCCTTGTTGATGTGTCGTCTCA | CCCCCAGCTCATAATCAAGAAC |
| *WRKY82* | AGTGAAAAGTAGTGAAAATTCCAG | GTGCTAGTTTCAATTATTCTGCTTCGT |
| *PR1a* | GGAAGTACGGCGAGAACATC | TGGTCGTACCACTGCTTCTC |
| *PR1b* | AGAACTACGCCAGCCAGAGAAG | TTCTCGCCAAGGTTGTTCCG |
| *PR2* | GGCAGGTGAGAGTCTACGAGGAA | GCTGTCATCCGAGCTAAGTGTT |
| *PR3* | CGTGTCTGTGGAGAGCGTGGTC | TCGTCGTTGGTGCGGTCATTGG |
| *PR4* | AGTATGGATGGACCGCCTTCTGT | CTCGCAATTATTGTCGCACCTGTTC |
| *PR5* | CGGTGCCCGCTGACGAGGA | GACGTCGACGGTGCGGATGA |
| *PR10* | CACCATCTACACCATGAAGC | AGCACATCCGACTTTAGGAC |
| *NPR1* | GGCAGGTGAGAGTCTACGAGGAA | GCTGTCATCCGAGCTAAGTGTT |
| *PAL1* | TCGGCTGCGTATTCCTCA | AGTTGATGGGAAGGGGCT |
| *PAL2* | GCATCAGCTTCCAACTCG | GGTTTCGCACTCCATTACAGA |
| *PAL3* | CGCTGAGGCGTTTAAGATTG | GGCAAGGACAGCAAGAATG |
| *PAL4* | CTTCACAACAGCTAATCGAG | CGCACTCCATTTCAGTACCA |
| *PAL5* | TGCTGTCCGCCGTGTTCT | CCACCTCGTTCACCTTCTGC |
| *PAL6* | AGATTGAGGTCATCCGTG | GAACATGAGCTTACCGATC |
| *PAL7* | ATCGACATCCTCAAGCTCATG | AGTTGGTGCTCAGCGTCTTCT |
| *EDS1* | CATTCCAAGAACGAGGACACTG | CAAGACTCAAGGCTAGAACCGA |

Additional file 1: Table S3: Markers used for fine mapping and sequencing of *spl40* locus

| Marker | Sense primer (5′–3′) | Antisense primer (5′–3′) |
| --- | --- | --- |
| RM17952 | TTTCCATTTCAGAGCAGGTTCC | GGGATGGTGAATTCCTTCTTGC |
| RM18360 | TCGAGACTGATCGGAGTTTAGGC | CGCTCCTCCCTAACACCTCTACG |
| RM18379 | GAGGTATGCTGCCTTATGCTCTGC | CTCAACTGCTCGCTCTCGATGC |
| RM18522 | ACCTCCTCGCTCGTCTCTCTCC | CCTGCCTCGTGAAGTTGAGAGC |
| InDel1 | CCTCGTTAGATTCGTCTCGCA | GCTCAGTTATTAGGTACTGTGTCG |
| GT | TGGTGCTTAAGTTGCTGTCT | AGTACCATGCCCGTAGCTTT |

Additional file 1: Table S4: Primers used for making complementation construct

| Marker | Sense primer (5′–3′) | Antisense primer (5′–3′) |
| --- | --- | --- |
| Frag1 | GGTACCTGAAGCACTGTGGACACCTTT | TATTCGGCGGTGGATTAGGG |
| Frag2 | ACGCCTGCCGTTCGACGATTCAAAACCACTCGCCCCGTAA | ACGCGCGGATCTTCCAGAGATTAGAGTCTAGGGGGAGCAATGT |
| Frag3 | ACGCCTGCCGTTCGACGATTTGGCGGCGTCTAAGATTCTG | ACGCGCGGATCTTCCAGAGATTTCTTCAGCGTCTCGAGCTTC |
| Frag4 | CCTCCCCAGTTTATACTTCCGT | CTGCAGGCCAGATTGGCTACAGTGG |
