# Supplementary material for: Identification and characterization of a spotted-leaf mutant spl40 with enhanced bacterial blight resistance in rice
Source: Rice (N Y). 2019 Aug 24;12:68. doi: 10.1186/s12284-019-0326-6 (PMC6708518; doi:10.1186/s12284-019-0326-6)
Supplement: Supplementary file 2 — Figure S1. Frequency of WT SPL40 allele in the T1 generation of complementary plants. Wild type (W), intermediate type (I) and Lesion mimic type(L). Values are means ± SD of three biological repeats and re subjected to one way ANOVA followed by the Duncan multiple range test. Figure S2. Phenotype of spl40 and spl40NIP. (a) Phenotype of spl40; (b) Phenotype of spl40NIP. (PDF 255 kb) [file 12284_2019_326_MOESM2_ESM.pdf]

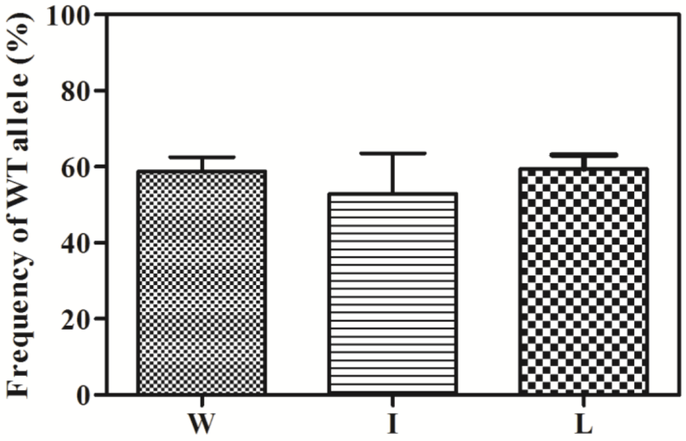

Figure S1. Frequency of WT *SPL40* allele in the T_1 generation of complementary plants. Wild type (W), intermediate type (I) and Lesion mimic type (L). Values are means ± SD of three biological repeats and are subjected to one-way ANOVA followed by the Duncan multiple range test.

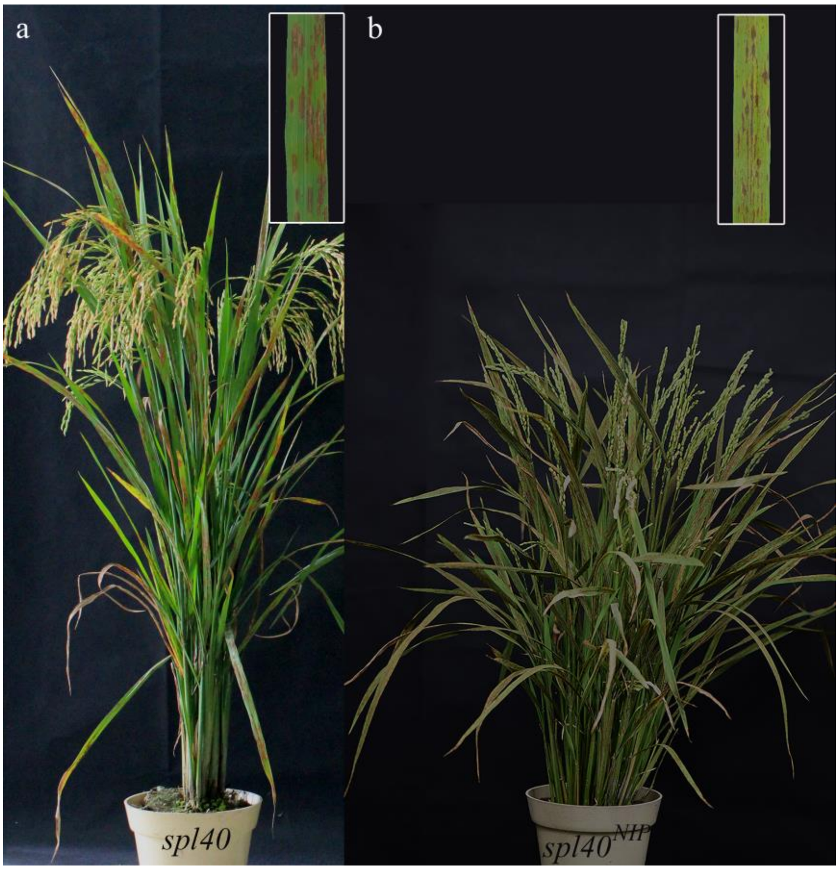

Figure S2 Phenotype of *spl40* and *spl40*^{NIP}. (a) Phenotype of *spl40* ; (b) Phenotype of *spl40*^{NIP}
